# An Improved Mixture-of-Gaussians Background Model with Frame Difference and Blob Tracking in Video Stream

**DOI:** 10.1155/2014/424050

**Published:** 2014-04-10

**Authors:** Li Yao, Miaogen Ling

**Affiliations:** Computer Science and Engineering Department, Southeast University, Nanjing 211189, China

## Abstract

Modeling background and segmenting moving objects are significant techniques for computer vision applications. Mixture-of-Gaussians (MoG) background model is commonly used in foreground extraction in video steam. However considering the case that the objects enter the scenery and stay for a while, the foreground extraction would fail as the objects stay still and gradually merge into the background. In this paper, we adopt a blob tracking method to cope with this situation. To construct the MoG model more quickly, we add frame difference method to the foreground extracted from MoG for very crowded situations. What is more, a new shadow removal method based on RGB color space is proposed.

## 1. Introduction

Detection and segmentation of moving objects in video streams are the first relevant step of information extraction in many computer vision applications, such as video surveillance, traffic monitoring, crowd counting, and people tracking. Mixture-of-Gaussians (MoG) background model is widely used in such application to segment moving foreground for its effectiveness in dealing with gradual lighting changes and repetitive motion of leaves.

However, the MoG method has two apparent shortages: one is slow construction of background model at the beginning and the other is that it cannot cope with the case that the objects enter the scenery, stay for quite a while, and leave, which often happens in subway, bus station, railway station, and so forth. When the objects stay longer, they would gradually merge into the background, which would affect the follow-up application, such as crowd counting or event analysis. Our objective is to find a method to solve these two crucial problems.

### 1.1. Related Work

Segmenting moving objects from video stream has been researched for a long time. The traditional method of averaging the image pixels over time to create a background is only effective in situations where the background is visible in a large proportion of the time.

Cucchiara et al. [1] proposed the median method, finding the median value for each pixel in certain nearest frames and the recorded median value as to eliminate the short peak values which affect the mean value a lot. However it has large time and space complexity.

The mixture-of-Gaussians approach [2] has gained tremendous popularity due to its capability to model multimodel backgrounds. It can deal with gradual lighting changes, repetitive motion of leaves, and so forth. It models each pixel as a mixture of Gaussian distributions but has a slow speed of background construction. KaewTraKulPong and Bowden [3] adopt an online K-means method approximation to update the model. They set different updating rate in background construction period and stationary period. At the beginning the updating rate is the reciprocal of frame number so as to speed up the establishment of MoG. Then after certain number of frames (100) the rate is set to a small value, such as 0.01, to ensure its stability. The updating rate of Gaussians in stationary period decides how fast it would adapt to illumination changes or a new background. The Gaussian distributions of the constructed background model are sorted according to their weights to determine which are most likely to be the background. The pixels which do not match the Gaussian distributions are regarded as foreground and a new Gaussian distribution with mean value of the pixel is established to replace the lowest weight distribution. Commonly speaking, 3 to 5 Gaussian distributions are adopted.

Wang et al. [4], Zongbin [5], and Yuan et al. [6] use different ways to improve the MoG method. Considering the effect of large areas of illumination changes, Wang et al. [4] replace the most probable Gaussian distribution with current pixel value when the foreground pixel numbers count for more than 60 percent of area of the whole image. Zongbin [5] divides the extracted foreground from MoG into moving objects and false positive pixels according to difference between current frame and the former frame. It updates the MoG model faster for false positive pixels which have lower frame difference while updating the moving objects slower which have large frame difference. It can adapt faster to sudden lighting changes for it updates the low frame difference pixels of the foreground which are caused by the changed lighting condition faster. Yuan et al. [6] use the similarity of interframe gradient to decide whether it is foreground or background for the gradient information varies slightly while the illumination changes. They propose a relevant function to calculate the similarity of gradient between current frame and former frames of certain gap. If the time of similarity is larger than a certain number, it would be added into the background. Zongbin [5] and Yuan [6] both effectively improve the performance of MoG in sudden lighting changes condition but they still ignore a commonly happening situation that a person came in, stayed for a while, and left. As the person stays longer, the person will be gradually merged into the background and could not be detected which may be undesirable for foreground extraction. If the person left, a “ghost” would be detected and last for a period of time.

Cuevas et al. [7–9] use a nonparametric modeling and a particle filter tracking for moving object detection. The background is modeled using only color information and the foreground combines both color and spatial information. The application of a particle filter allows the update of the spatial information and provides a priori information about the areas to analyze in the following images, enabling an important reduction in the computational requirements and improving the segmentation results. Cuevas et al. [10] apply the algorithm to a general purpose graphics processing unit (GPGPU), which provides real-time and high-quality results in a great variety of scenarios.

### 1.2. System Overview

**Figure 1:**
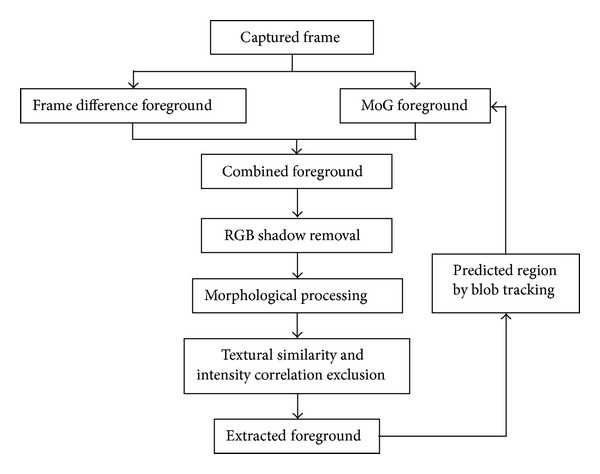
Flow chart of the proposed method.

As shown in Figure 1, we use frame difference method and the traditional MoG method to extract foreground for each frame. Then a shadow removal method based on RGB color space is adopted to detect shadow. And then we use a morphological method to connect the separate part of one object. A method based on texture similarity and intensity cross-correlation method is used to detect illumination change. We use a blob tracking method to predict successive blob in next frame and merge it into the MoG method to help speed up modeling. Finally we acquire the extracted foreground.

Our background modeling algorithm provides the following contributions.

- We add frame difference to MoG method to speed up the initial construction of MoG model.
- We use blob tracking to help MoG method to cope with the situation that the objects come in, stay for a while, and leave.
- We propose a simple sole blob extraction method.

## 2. Adding Frame Difference to MoG in Crowded Situation

MoG method needs several frames to construct a stable background at the beginning. Usually in uncrowded situation, 10 to 20 frames are enough in a 10 fps video. However, in very crowded situations, the background could hardly be seen; it will last much longer to form the right background model using the original MoG method. Sometimes as the background is shaded by the walking people in most of the time, the constructed background is not complete and the foreground would be badly extracted.

Thus we consider a three-frame difference method [11] as the compensation for foreground extraction especially in crowded situation where the objects moving slowly in the distance or the background could hardly be seen for most of the time. Surely we notice that if we do so, every object in foreground will be thicker. Thus we use a shadow removal technique to reduce the surrounded unnecessary pixels.

We first calculate the difference image between every three successive frames, using a certain threshold to get a binary, black-and-white image to have a coarse detection of foreground. Then we add the result to the foreground extracted from MoG to obtain the right foreground much faster. Median filtering is used to remove the unnecessary noise points. And with the following blob tracking method, we construct the right background model in MoG fast.

## 3. Shadow Removal in RGB Color Space

**Figure 2:**
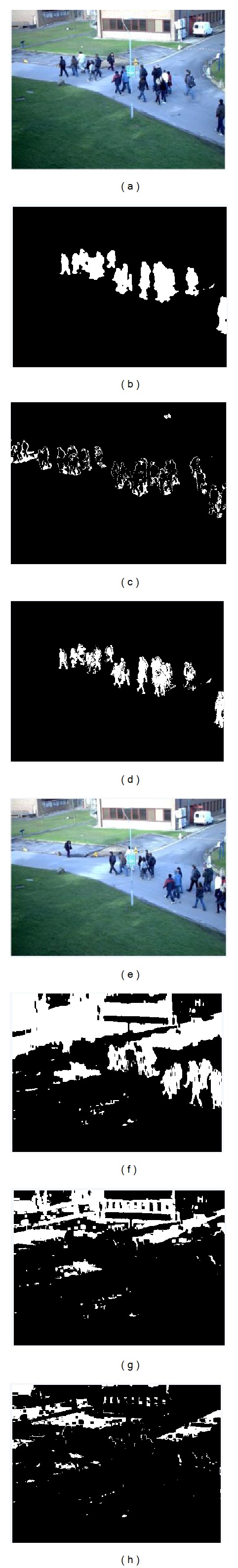
Two frames and the results with shadow removal. (a) and (e) are the frames (numbers 065 and 038) from the PETS2009 S1L1 with crowded people, (b) and (f) are the results of MoG method with frame difference, (c) and (g) are the detected shadows, and (d) and (h) are the results after removing the shadow.

We use a new way to detect shadow in RGB color space, inspired by the method in HSV color space by Cucchiara at al. [12, 13]. We notice the green component of a shadowed point is a bit lower than that of the background, so we first set two thresholds for the rate of pixels between current image and background image. What is more, the red and blue components of a pixel do not change significantly when a shadow is cast and the blue component is lowered in shadowed points. Consequently, the decision process is based on the following equation:

(1) $$\begin{array}{c} S_{k}\left(x,y\right)=\{\begin{array}{cc} 1 & \text{if}\;\alpha \leq \frac{I_{k}^{G}\left(x,y\right)}{B_{k}^{G}\left(x,y\right)}\leq \beta \\ & \wedge \left|I_{k}^{R}\left(x,y\right)-B_{k}^{R}\left(x,y\right)\right|<\gamma \\ & \wedge \left|I_{k}^{B}\left(x,y\right)-B_{k}^{B}\left(x,y\right)\right|<\gamma \\ & \wedge I_{k}^{B}\left(x,y\right)<B_{k}^{B}\left(x,y\right)\\ 0 & \text{otherwise}, \end{array} \end{array}$$

where *I*_*k*_(*x*, *y*) are the pixel values for the extracted foreground and *B*_*k*_(*x*, *y*) for the background. If *S*_*k*_(*x*, *y*) = 1 the pixel is assumed to be covered by a shadow. *α* should be adjusted according to the strength of the light source causing the shadows, *β* is needed to cope with certain aspects of noise, and *γ* is a threshold which decide how large the difference in red and blue component can be. We choose *α* = 0.75, *β* = 0.98, and *γ* = 50 in our experiments. The shadow result is showed in Figure 2(c) for example.

A morphological close operation is adopted to connect the separated parts and fill small holes inside the foreground caused by RGB shadow removal. We use a 5x1 structural element for it may connect the upper and lower body of a person and avoid connecting two adjacent persons.

## 4. Texture and Intensity Integration for Quick Lighting Changes

The mixture-of-Gaussians method generates large areas of false positive foreground when there are quick lighting changes (Figure 2(f)). To make the mixture-of-Gaussians method work for quick lighting changes, we adopt the method by Tian et al. [14], integrating the texture information to the foreground mask for removing the false positive areas. The basic idea is that the texture in the false positive foreground areas which is caused by lighting changes should be similar to the texture in the background. The erroneously detected foregrounds from texture similarity are shown in Figure 2(g).

The intensity information is employed instead of color information in shadow removal. The normalized cross-correlation of the intensities is calculated at each pixel of the foreground region between the current frame and the background image by Tian et al. [14]. The detected shadows are showed in Figure 2(h), which is a good compensation of texture similarity detection.

## 5. Blob Tracking Method

The original and improved MoG methods [2, 3] update the background models for every pixel in a frame. Apparently, the foreground pixels are useless in background modeling and if the foreground pixels stay longer, it would be merged into the background, causing the missing detection. And then if the foreground pixels leave their original position, it would cause “ghost” in the extracted foreground for quite a long time.

In this paper, we use blob tracking method to solve the problem. First we could get diverse blobs of foreground in video streams for the former two frames using the original MoG method. Then we try to find matches in blobs between the two frames and use the matching information to predict the blob's position in the current frame. We combine all the predicted blobs to form a prediction of the current frame. Then we greatly slow down the updating speed in MoG in pixels of the predicted frame, which means we give a very low learning rate for weights, means, and variances of MoG of those pixels to prevent them from merging into the background.

### 5.1. Sole Blob Extraction in Foreground

We scan line by line to find the first white pixel and then search right if the right pixel or the second right pixel is white. Each time we reach a searching position, we make it black to prevent it from repetitive searching. We store the leftmost and rightmost position, and search the white pixel in the next line from leftmost to rightmost, and search left and right more as above until it reaches the new leftmost and rightmost position. If the new rightmost position is bigger than the last line for more than two pixels, we will search upward line by line for the exceeded pixels and also search left and right more until we meet a line with no white pixel. The new leftmost position would do the same way. Using this method, we may easily get the sole blob from the foreground. The pseudocodes are as in Algorithm 1.

### 5.2. Blob Tracking Method

After saving all the blobs in the former two frames, the “direct match method” [15] is adapted to detect direct matches between overlapping blobs in the former two frames.

We use the saved coordinates to calculate the center of each blob and compare the distances of centers between blobs in two frames with a certain threshold. The two blobs whose distances are under the threshold, which means they are very close, will be considered in the overlapping judgment.

Given regions *A* and *B*, let *O*(*A*, *B*) denote the fraction of region *A* that overlaps *B:*

(2) $$\begin{array}{c} O\left(A,B\right)=\frac{\left|A\cap B\right|}{\left|A\right|}. \end{array}$$

Let *B*_*t*,*m*_ denote the *m*th blob in frame *t*. Blobs are compared between frame *t* and *t* + 1 in order to attempt the matching of blob *B*_*t*,*m*_ to blob *B*_*t*+1,*n*_. This is done by calculating two overlaps: forward overlap *F*_*t*_(*m*, *n*) and reverse overlap *R*_*t*_(*m*, *n*) which are calculated by

(3) $$\begin{array}{c} F_{t}\left(m,n\right)=O\left(B_{t,m},B_{t+1,n}\right),\\ R_{t}\left(m,n\right)=O\left(B_{t+1,n},B_{t,m}\right). \end{array}$$

To match blobs *B*_*t*,*m*_ and *B*_*t*+1,*n*_, it is necessary to ensure sufficient overlap

(4) $$\begin{array}{c} F_{t}\left(m,n\right)\geq T_{\min},\\ R_{t}\left(m,n\right)\geq T_{\min}. \end{array}$$

To distinguish a match from the split or merge events, this overlap should be mostly exclusive to *B*_*t*,*m*_ and *B*_*t*+1,*n*_. Therefore, the following requirements are also needed:

(5) $$\begin{array}{c} F_{t}\left(i,n\right)\geq T_{\max}\;\forall i\neq m,\\ R_{t}\left(i,n\right)\geq T_{\max}\;\forall i\neq m,\\ F_{t}\left(m,j\right)\geq T_{\max}\;\forall i\neq n,\\ R_{t}\left(m,j\right)\geq T_{\max}\;\forall i\neq n. \end{array}$$

Any blob pair (*m*, *n*) which satisfies conditions (4)-(5) is considered as a match. The threshold values *T*_min⁡_ and *T*_max⁡_ are used to filter out the false matches. The values *T*_min⁡_ = 0.5 and *T*_max⁡_ = 0.2 were selected.

**Figure 3:**
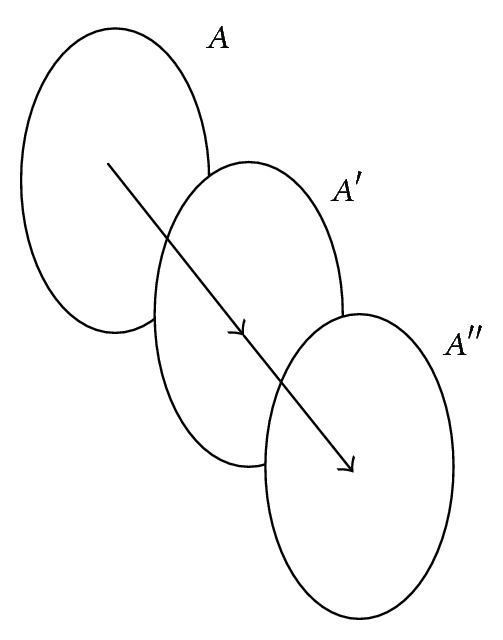
Example of blob prediction from the matched blob in the previous two frames.

Then we use the two matched blobs (one in each frame) to predict the current position of this blob (Figure 3). We only need calculate the approximate move vector $\overset{⃑}{M}=C_{A^{\prime }}-C_{A}$, where *C*_*A*′_ and *C*_*A*_ mean the center coordinates of blob *A* and *A*′. And then we move the blob *A*′ along $\overset{⃑}{M}$ to get the predicted position of blob *A*′, which is *A*′′  as shown in Figure 3.

Our method may sharply reduce the updating rate to prevent the moving blob from merging into the background, which will surely keep the right background much longer. Especially if we encountered a person who entered the scene, stayed for quite a while, and left, our method has an excellent performance.

Then, we explain the new updating strategy of weights, means, and variances of MoG models.

The original weights of *K* Gaussian distributions at time *t*, *w*_*k*,*t*_, are adjusted as follows:

(6) $$\begin{array}{c} w_{k,t}=\left(1-\alpha \right)w_{k,t-1}+\alpha \left(M_{k,t}\right), \end{array}$$

where *α* is the learning rate and *M*_*k*,*t*_ is 1 for the model which matched and 0 for the remaining models.

Now we change it to

(7) $$\begin{array}{c} w_{k,t}=\left(1-\alpha^{\prime }\right)w_{k,t-1}+\alpha^{\prime }\left(M_{k,t}\right),\;\alpha^{\prime }=\frac{\alpha }{C} \end{array}$$

for the predicted region of the current frame and *C* is a constant as 50 or larger.

The *μ* and *σ* parameters, which represent mean and variance of Gaussian, are unchanged for unmatched distributions. The pixels which match the new observation are updated as follows:

(8) $$\begin{array}{c} \mu_{t}=\left(1-\rho^{\prime }\right)\mu_{t-1}+\rho^{\prime }X_{t},\\ \sigma_{t}^{2}=\left(1-\rho^{\prime }\right)\sigma_{t-1}^{2}+\rho^{\prime }{\left(X_{t}-\mu_{t}\right)}^{T}\left(X_{t}-\mu_{t}\right), \end{array}$$

where *ρ*′ = *ρ*/*C*. *ρ*, the updating rate, is set to 0.01 (or 0.005) after 100 (or 200) frames. *X*_*t*_ is the current pixel value at time *t*.

Thus we can remove the unnecessary pixels of moving objects from merging into the background effectively, when the moving object stays for a while, and avoid the “ghost” after its leaving.

What is more, our method can help in constructing the background much faster when dealing with very crowded situation for it has cut down lots of unnecessary moving blobs to join the background of MoG which count a lot at the beginning period of modeling.

## 6. Experimental Results

We use PETS2009 database [16] to test our algorithm for the people in the sceneries which are more crowded. And then we use a video of our own to test the situation that one comes into the scenery and stay for a while.

### 6.1. Comparison in Crowded Situation in the Distance

In the crowded situation, our method only needs about 30 frames to form a stable background.

**Figure 4:**
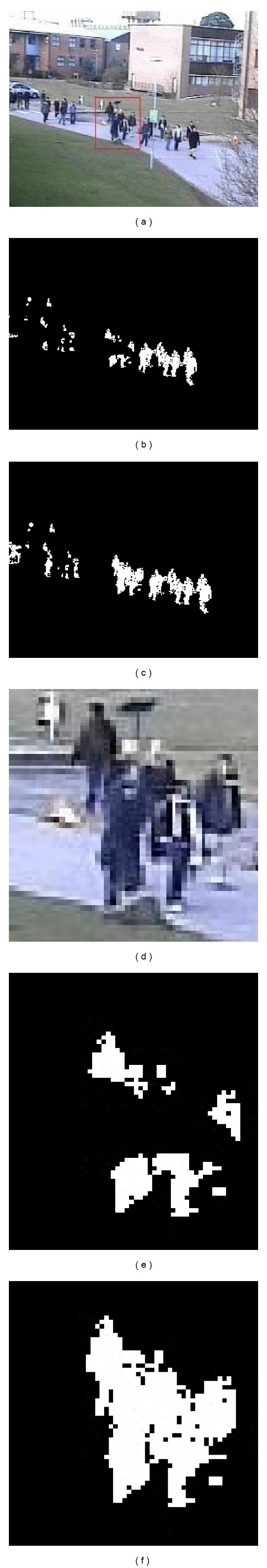
Comparison of MOG and “MOG with blob tracking”: (a) a frame (number 094) from the video with crowded people, (b) result of original MoG, (c) result of MoG with blob tracking, and (d), (e), and (f) in the bottom are the detail of center part of the above images.

In Figure 4, we compare the original MoG with the MoG combining blob tracking (both without frame difference). It is obvious that the method with blob tracking gets much better performance in the extracted foreground for we exclude lots of unnecessary moving points in background updating.

**Figure 5:**
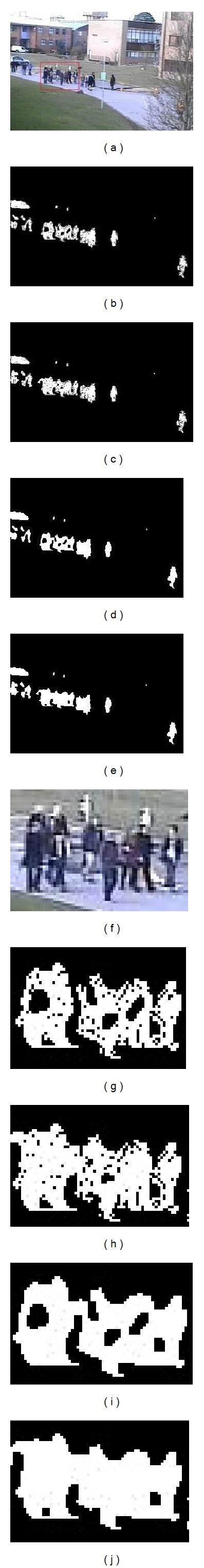
Comparison of “MoG with frame difference” and “MoG with blob tracking and frame difference”: (a) a frame (number 050) from the video with crowded people, (b) result of original MoG with frame difference, (c) result of MoG with blob tracking and frame difference, (d) and (e) results after a close operation of (b) and (c) separately, and (f), (g), (h), (i), and (j) in the bottom are the detail of center part of the above images.

In Figure 5, we compare the original MoG with the MoG combining the blob tracking (both with frame difference). We can see that the blob tracking helps to extract the moving objects more completely than the original MoG with frame difference, even after a close operation. Figures 5(i) and 5(j) are the images resulting after a close operation to connect the adjacent components to form a whole blob. As we can see, results from the MoG only with frame difference have big holes inside which are not easy to fill while our method has very small leave-outs and acquires much better results after the close operation.

### 6.2. Comparison for One's Stay for a While

In indoor sequences we do not need frame difference to help construct the background but only the tracking method.

**Figure 6:**
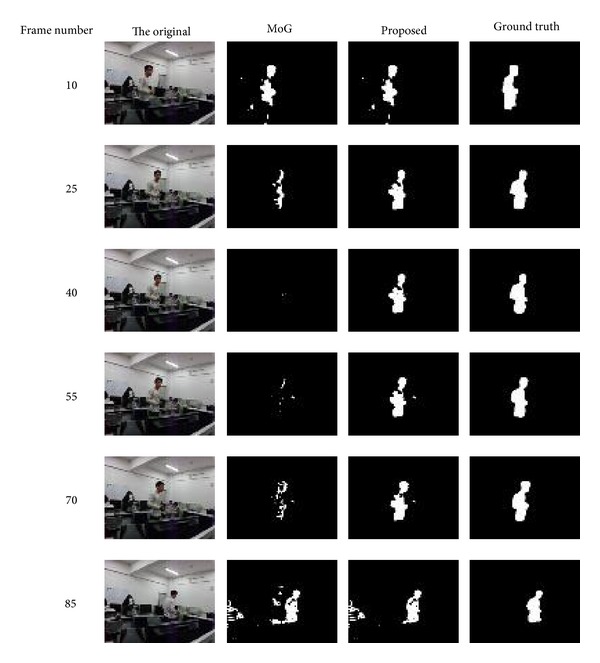
Examples of foreground segmentation results comparing the proposed method with the original MoG.

We recorded a video to test the foreground extraction in the “came into the spot, stayed for a while, and left” situation. In the video one person walked from one side to the other side of the room and then walked back, remaining in the middle of the room for 3-4 seconds. Its frame rate is 10 fps. For the first 10 frames we only use MoG to get the initial background. After that, we add blob tracking to MoG method. The initial FNRs and FPRs of foreground extraction are shown in Table 1 and several foreground segmentation results in Figure 6. We can see that people's staying for a while affects the foreground a lot especially at the initial construction period which has high updating rate of Gaussian model. After the initial construction period, people's stay will cause less and slower influence, depending on the updating rate but the extracted foreground will also decrease as one's prolonged stay.

At the initial construction period, only 3-4 seconds' stay of people would cause loss of large area of foreground in the original MoG, such as 40th frame of MoG in Figure 6. It can be seen that spurious foreground regions disappear after only about 3 seconds' stay. However in our method, most parts of moving person are preserved almost along the whole modeling procedure. After simple morphological processing, recovering some missing parts caused by the color similarity of foreground to the background, our method's segmented foregrounds are very close to the ground truth foreground.

## 7. Conclusion

The algorithm performs nearly real time. Our method needs 0.15 s averagely for each frame (nearly 7 frames a second) with a 2.4 GHz CPU in Win7. Our method successively solved the problem of person's “came in, stayed for a while, and left” in a video sequence, which is quite common in public sceneries. And with the blob tracking method, we could construct the background much more soon and extract more accurate foreground.

The proposed method performs much better than the original MoG method. However, we should also notice that it could not cope with the situation that objects in background from the start are later moved away, which will cause a ghost subsequently. When dealing with fast moving objects, such as moving cars, the blob tracking method should be replaced by a speed insensitive method. The simplization of the MoG method to satisfy real-time application also needs to be considered later.

## Figures and Tables

**Algorithm 1:**
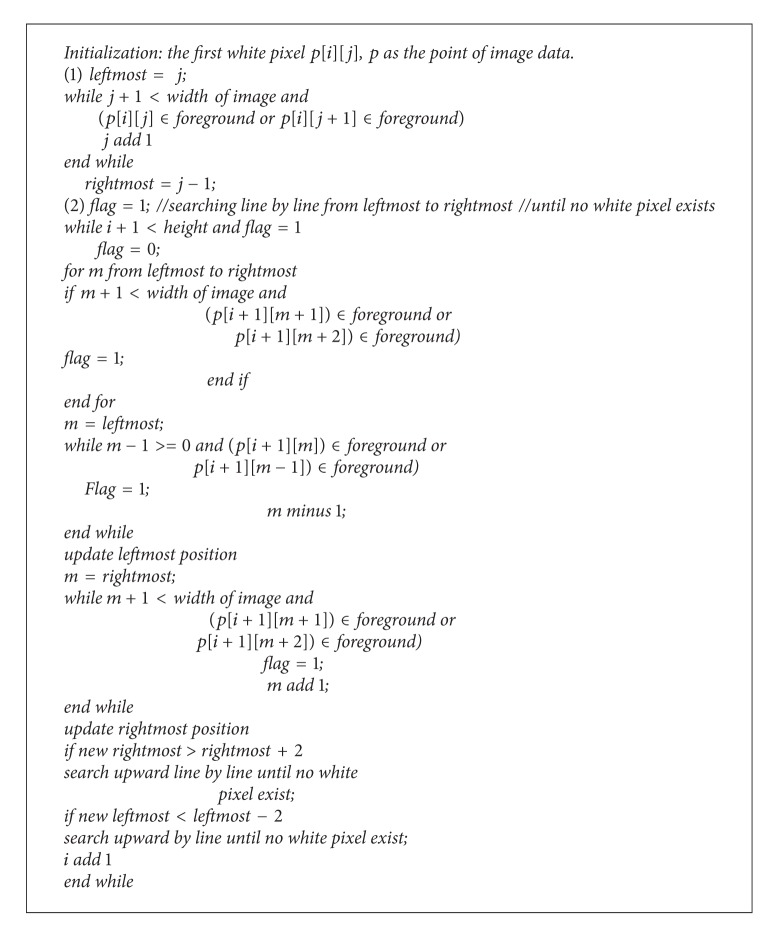
Pseudocode for sole blob extraction.

**Table 1:** FNRs and FPRs (%).

Method	My video			
	Foreground after 60 frames		Foreground after 100 frames	
	FNR	FPR	FNR	FPR
MoG	61.8	0.2	56.3	0.6
MoG + blob tracking	13.1	0.6	12.7	0.8
